# 1786. Separating the Rash from the Chaff: Novel Clinical Decision Support Deployed During the Mpox Outbreak

**DOI:** 10.1093/ofid/ofad500.1615

**Published:** 2023-11-27

**Authors:** Jacob E Lazarus, Chloe V Green, John Albin, Caitlin Dugdale, Kevin L Ard, Lindsay Germaine, Elizabeth L Hohmann, Kristen Hysell, Dustin S McEvoy, Hang Lee, Erica S Shenoy

**Affiliations:** Massachusetts General Hospital, Boston, Massachusetts; Massachusetts General Hospital, Boston, Massachusetts; Massachusetts General Hospital, Boston, Massachusetts; Massachusetts General Hospital, Boston, Massachusetts; Massachusetts General Hospital, Boston, Massachusetts; Mass General Brigham, Portland, Maine; Mass General Hospital, Boston, Massachusetts; Massachusetts General Hospital, Boston, Massachusetts; Mass General Brigham, Portland, Maine; MGH, Boston, Massachusetts; Massachusetts General Hospital, Boston, Massachusetts

## Abstract

**Background:**

The mpox outbreak of 2022 posed significant challenges to clinicians: how to identify individuals with potential infection and then take appropriate next steps, all in the context of an emerging infection with which they were unfamiliar. By facilitating adaptable, guideline-concordant management, clinical decision support systems (CDSS) have been shown to support clinicians in such efforts.

**Methods:**

We integrated the “Evaluate for mpox” (.evalmpox) CDSS into the EHR (Epic Systems) in a large, integrated healthcare system. .evalmpox was designed to facilitate identification of patients with epidemiological risk factors warranting Person Under Investigation (PUI) status (Figure 1). We previously described our CDSS prototype and the evaluation of 55 patients through 7/20/2022. Here, we describe substantive tool enhancements including improved symptom collection, patient triage, and automated isolation and de-isolation workflows (Figure 2). We also report use characteristics and tool performance through 4/13/2023.

**Figure 1.**
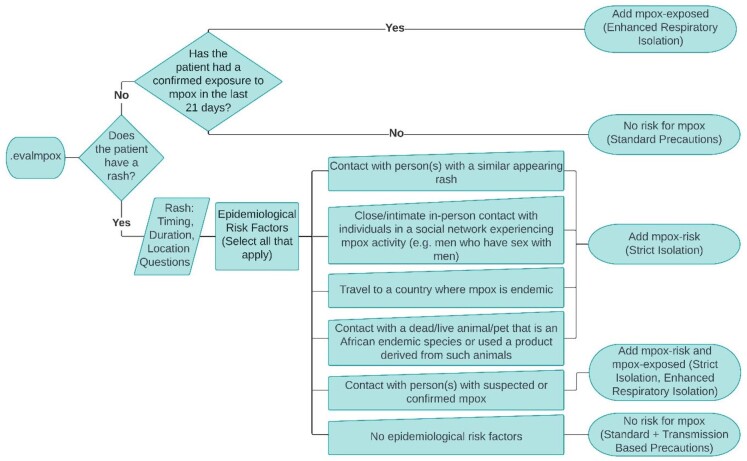
evalmpox CDSS logic diagram.evalmpox facilitates identification of patients with rash and epidemiological risk factors warranting mpox PUI status, or conversely for those without risk factors, rapid mpox rule-out. .evalmpox also coordinates automatic infection control isolation status (at the top of figure, Enhanced Respiratory Isolation for those exposed to mpox, and below, Strict Isolation for those meeting PUI criteria).

**Figure 2.**
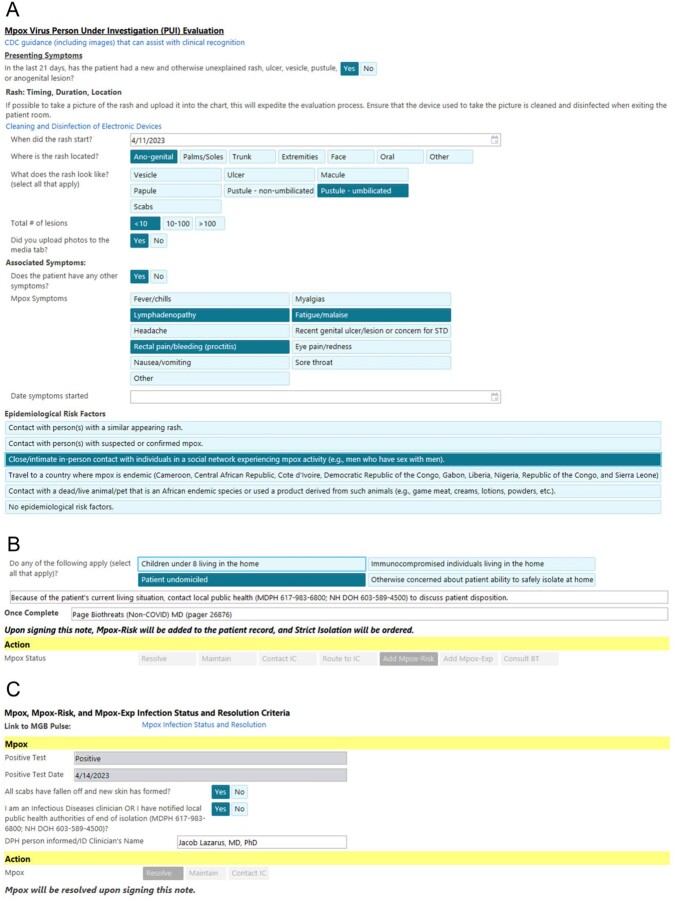
evalmpox augmented versioning. A) From top, .evalmpox guides clinicians to sample images of mpox rashes and guides history taking to allow standardized collection of information on rash onset, location, qualities, and associated systemic symptoms. It also prompts the clinician to document the rash photographically to assist in the evaluation of rash evolution over time. This standardized approach also accomplishes clinician teaching on features of this unfamiliar disease, and ensures evaluation for signs or symptoms that may not be part of a routine evaluation (e.g. pharyngitis, proctitis). Risk factor identification assists with contact tracing. B) By collecting information on challenges to discharge home, .evalmpox allows early involvement of in-house case management and Department of Public Health input. C) .evalmpox also allows resolution of mpox infection control isolation statuses once the rash has resolved.

**Results:**

.evalmpox was used in 721 encounters (Figure 3) originating from 114 clinical locations, with 262, 249, 185, and 25 from the ED, outpatient, urgent care, and inpatient settings. 650 total non-duplicate patients were evaluated, with 265 classified as PUIs based on rash and the presence of at least one epidemiological risk factor, and 385 as non-PUI based on the absence of reported risk factors (Figure 4). Among 265 PUIs, 185 had mpox testing performed: 103 (57%), 69 (36%), 10 (5%), and 3 (2%) were negative, positive, indeterminate, or unable to be run. Among the 385 non-PUIs, 49 had mpox testing: 43 (88%), 4 (8%), and 1 (2%) were negative, positive, and indeterminate. The PPV of .evalmpox for a positive test in our EHR was 38% (95% CI 31%-45%) and the NPV was 99% (95% CI 97%-100%). Chart review revealed two of the four patients classified as non-PUI by .evalmpox who tested positive for mpox reported risk factors to clinicians that were not input into the tool.

**Figure 3.**
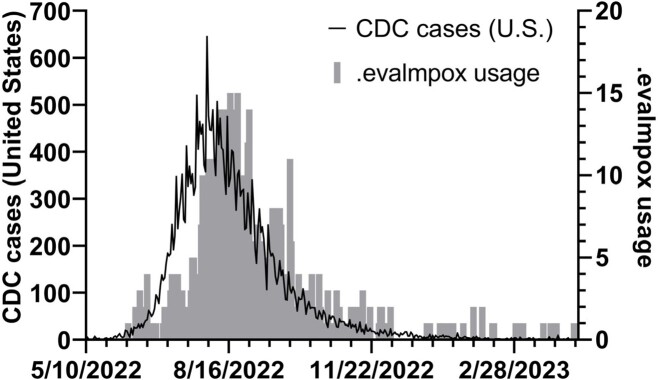
US mpox cases and .evalmpox utilization. .evalmpox facilitated the evaluation of patients with possible mpox in 721 encounters (as of 4/13/2023). Peak mpox cases reported to CDC on 8/1/2022, and CDSS use peaked on 8/17/2022. Peak positive mpox tests were sent on 8/2/2022 from Mass General Brigham (not depicted).

**Figure 4.**
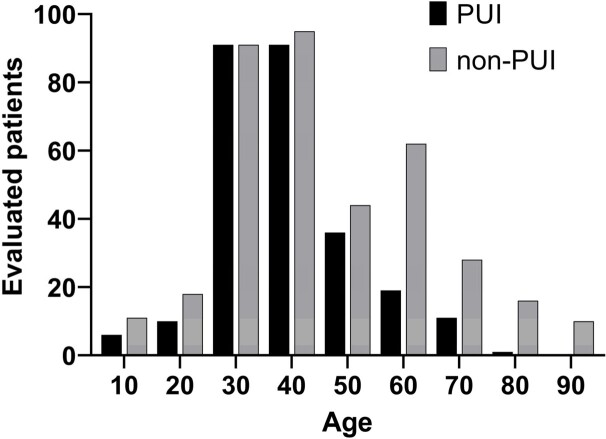
Age distribution of patients evaluated for mpox (in ten-year bins). Patients classified as PUI by .evalmpox had a peak in those aged in their 30's, similar to CDC data. Patients classified as non-PUI were older than those classified as PUI.

**Conclusion:**

In the setting of an evolving outbreak, .evalmpox facilitated the evaluation of patients presenting with possible mpox across a large healthcare system in diverse clinical locations and performed with favorable test characteristics.

**Disclosures:**

**Jacob E. Lazarus, MD, PhD**, SEED Biologics: Advisor/Consultant|UpToDate: Advisor/Consultant **Kevin L. Ard, MD**, Binx Health: In-kind research support **Elizabeth L. Hohmann, M.D.**, gilead: Advisor/Consultant|Kowa Pharmaceuticals: Advisor/Consultant

